# Identification of the haemodynamic environment permissive for plaque erosion

**DOI:** 10.1038/s41598-021-86501-x

**Published:** 2021-03-31

**Authors:** Michael McElroy, Yongcheol Kim, Giampaolo Niccoli, Rocco Vergallo, Alexander Langford-Smith, Filippo Crea, Frank Gijsen, Thomas Johnson, Amir Keshmiri, Stephen J. White

**Affiliations:** 1 Department of Mechanical, Aerospace and Civil Engineering (MACE), The University of Manchester, Manchester, M13 9PL UK; 2 Division of Cardiology, Department of Internal Medicine, Yonsei University College of Medicine and Cardiovascular Center, Yongin Severance Hospital, Yongin, Republic of Korea; 3 Division of Cardiology, Department of Medicine and Surgery, University of Parma, Parma, Italy; 4 Fondazione Policlinico Universitario A. Gemelli IRCCS, Rome, Italy; 5 Universita’ Cattolica del Sacro Cuore, Rome, Italy; 6 Department of Life Sciences, Manchester Metropolitan University, Manchester, M1 5GD UK; 7 Department of Cardiology, Erasmus Medical Centre, Rotterdam, The Netherlands; 8 Department of Biomechanical Engineering, TUDelft, Delft, The Netherlands; 9 Department of Cardiology, Bristol Heart Institute, University Hospitals Bristol and Weston NHS Foundation Trust, Upper Maudlin St., Bristol, BS2 8HW UK

**Keywords:** Cardiology, Cardiovascular biology

## Abstract

Endothelial erosion of atherosclerotic plaques is the underlying cause of approximately 30% of acute coronary syndromes (ACS). As the vascular endothelium is profoundly affected by the haemodynamic environment to which it is exposed, we employed computational fluid dynamic (CFD) analysis of the luminal geometry from 17 patients with optical coherence tomography (OCT)-defined plaque erosion, to determine the flow environment permissive for plaque erosion. Our results demonstrate that 15 of the 17 cases analysed occurred on stenotic plaques with median 31% diameter stenosis (interquartile range 28–52%), where all but one of the adherent thrombi located proximal to, or within the region of maximum stenosis. Consequently, all flow metrics related to elevated flow were significantly increased (time averaged wall shear stress, maximum wall shear stress, time averaged wall shear stress gradient) with a reduction in relative residence time, compared to a non-diseased reference segment. We also identified two cases that did not exhibit an elevation of flow, but occurred in a region exposed to elevated oscillatory flow. Our study demonstrates that the majority of OCT-defined erosions occur where the endothelium is exposed to elevated flow, a haemodynamic environment known to evoke a distinctive phenotypic response in endothelial cells.

## Introduction

Atherosclerotic plaque disruption can trigger thrombosis, causing restriction or total occlusion of blood flow and myocardial ischemia. Within the coronary circuit, approximately 65% of acute coronary syndromes (ACS) result from plaque rupture, while 25–30% result from plaque erosion^1–4^. Significant scientific attention has progressed our understanding of plaque rupture, however, plaque erosion remains enigmatic. The histological features of atherosclerotic plaques that rupture or erode differ markedly, suggesting divergence in the underlying mechanisms, with distinct risk factors contributing to each process. For example, plaque rupture occurs on inflamed and lipid-rich plaques with thin fibrous caps, contrasting with erosion-prone plaques containing abundant smooth muscle cells and few resident leukocytes with an intimal extracellular matrix containing high levels of versican and hyaluronan, molecules implicated in altering endothelial function^1, 5–9^.

Optical coherence tomography (OCT)-based intravascular imaging can distinguish rupture from erosion in patients with ACS^10–12^. OCT-defined erosion is a diagnosis made by exclusion of rupture, or the presence of eruptive calcified nodules, and has engendered some controversy^13^. Nevertheless, OCT-defined erosion has a similar frequency to that defined by histological studies^2, 11^, and shares demographic features, including age, sex and smoking status, further supporting the relevance of OCT-guided diagnosis^2, 4, 7, 11, 14–16^. Yet, other traditional risk factors for ACS, including diabetes, hyperlipidaemia and hypertension, identify with plaque rupture, highlighting that the mechanisms of endothelial erosion differ from those of plaque rupture and require better understanding. These considerations mandate further mechanistic investigation of endothelial erosion.

The haemodynamic environment exquisitely regulates endothelial behaviour: it modulates metabolism, cell shape, cytoskeleton arrangement, proliferation, permeability, response to inflammatory stimuli and apoptosis^17–21^. Consequently, haemodynamics strongly influence plaque development/progression and the lesion characteristics that determine their propensity to rupture^22, 23^. Therefore we evaluated features of the haemodynamic environment permissive for clinically-relevant plaque erosion, to aid investigation of mechanisms that drive pathology.

## Results

### Analysis of the haemodynamic environment underlying adherent thrombi

**Figure 1:**
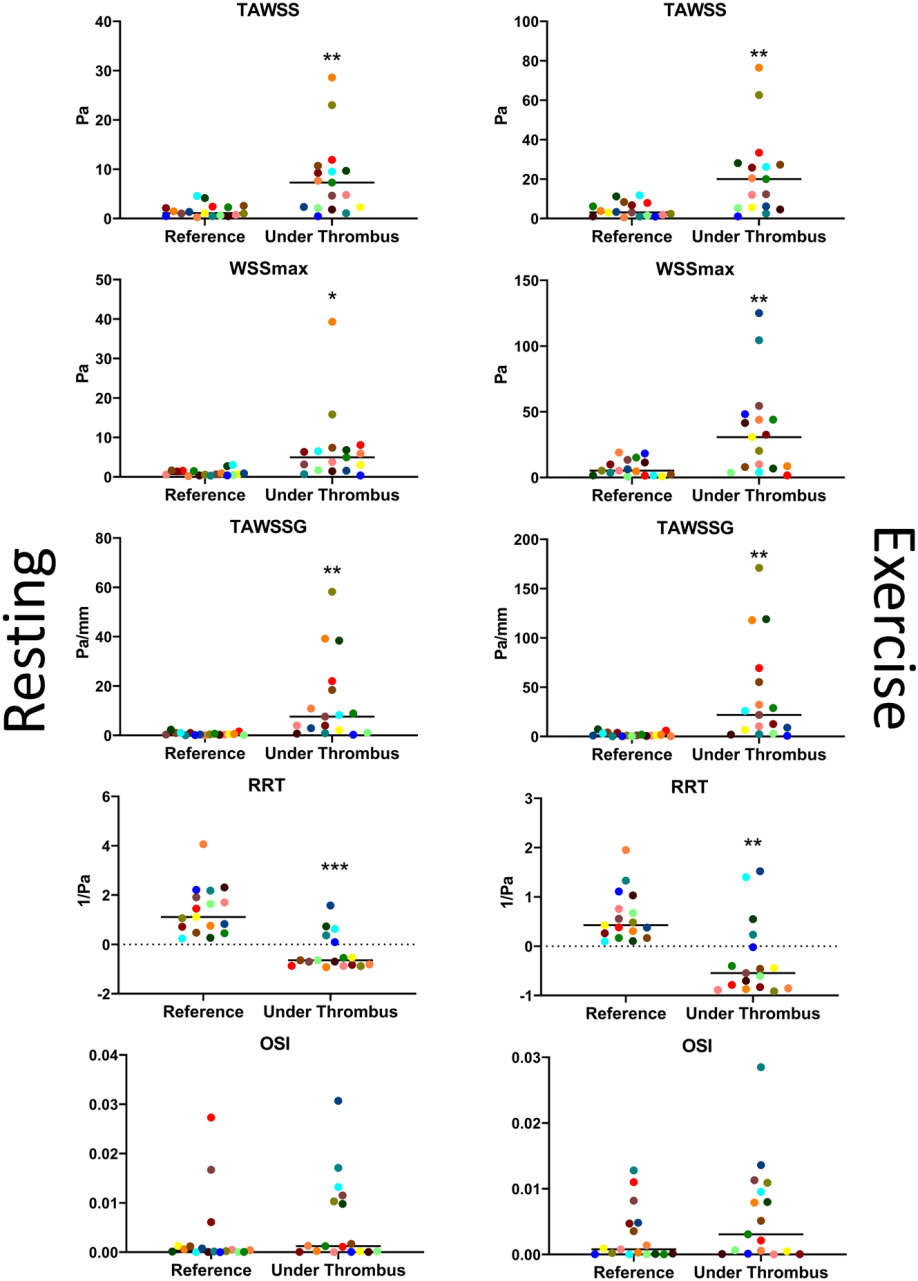
CFD analysis of artery wall underlying adherent thrombi. CFD was used to calculate the spatially averaged TAWSS within a non-diseased area and under the thrombus (n = 17) identifying a significant elevation of TAWSS, the median value is displayed on the graph (*p < 0.05, **p < 0.01, ***p < 0.001 paired T-test). No significant difference in OSI was observed between groups.

Our population was predominantly male (70.6%), with a mean age of 49 years and 64.7% were current or ex-smokers (Table 1). The majority of culprit plaques imposed a stenosis on the artery (diameter stenosis 30.9% [28.2, 51.9], area stenosis 52.3% [48.4, 76.9]; median and interquartile range) with 16 of the 17 cases having thrombi adhering proximal to, or overlying the point of maximum stenosis. Simulation of flow allowed the comparison of flow metrics between the sites of adherent thrombi (assumed to be synonymous with area of endothelial erosion) and an upstream non-diseased reference section. In line with the observed location of the thrombi on stenotic plaques, all metrics related to an elevation of flow were observed. These included an increase in spatially averaged time averaged wall shear stress (TAWSS ~ fivefold for both normal and exercise simulation), maximum wall shear stress (WSS_max_, 22 or 20-fold normal/exercise simulation respectively), spatially averaged time averaged wall shear stress gradient (TAWSSG, 6.5 or 5-fold normal/exercise simulation respectively) and reduced relative residence time (RRT, 4 or 2-fold normal/exercise simulation respectively) (Fig. 1). There was no significant increase in oscillatory shear index (OSI) between reference and area under thrombi. Thromboaspiration was not performed prior to imaging to reduce the likelihood of iatrogenic disruption of plaque morphology and subsequent difficulty in defining the location of the erosion site.

### Analysis of culprit plaque characteristics

**Figure 2:**
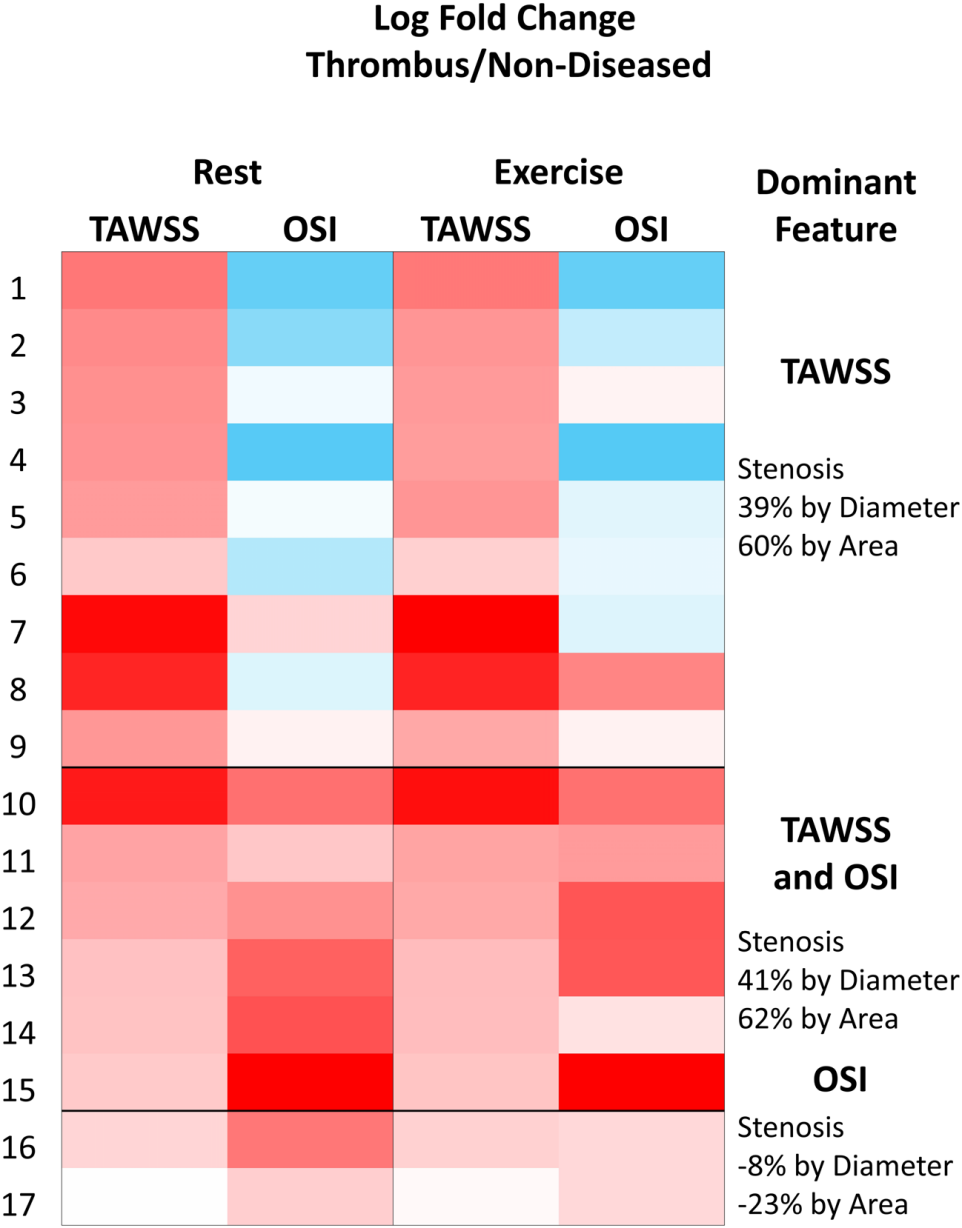
The log2 fold change displayed as a heatmap between the non-diseased reference segment and thrombus-covered areas, with red representing an increase and blue indicating a decrease.

**Figure 3:**
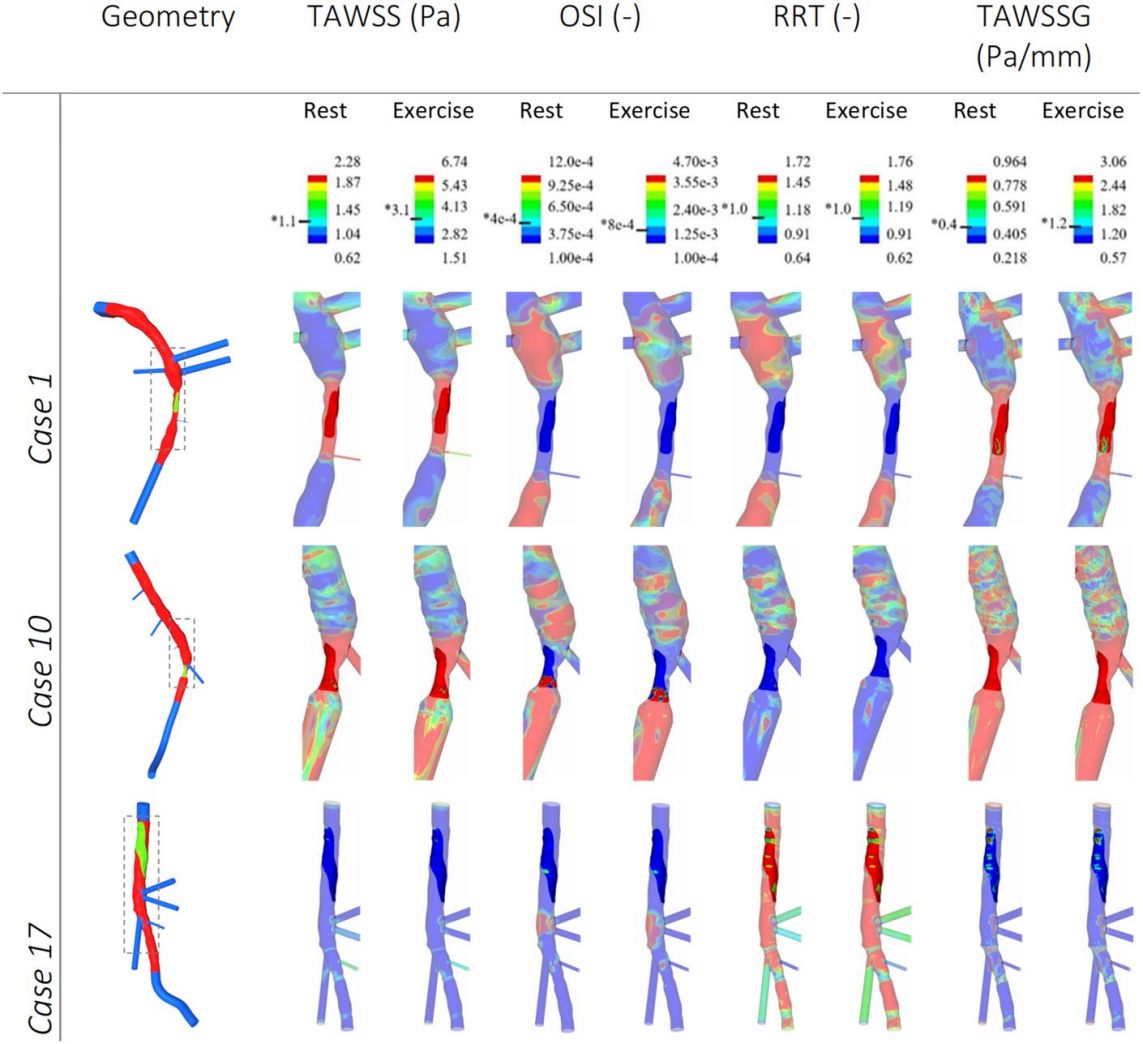
Reconstructed lumen geometries of the LAD, LCX and RCA arteries. Haemodynamic metrics were extracted from CFD simulations. Spatially averaged Time-Averaged Wall Shear Stress (TAWSS), Oscillatory Shear Index (OSI), Relative Residence Time (RRT) and Time-Averaged Wall Shear Stress Gradient (TAWSSG). Both ‘rest’ and ‘exercise’ flow rate conditions were simulated. The thrombus is the opaque portion of the metrics, whilst the remainder of the lumen is semi-transparent. Flow is from top to bottom for all images. Minimum and maximum values for the legends are the lower and upper quartiles of the respective metrics averaged across the rest and exercise cases separately, as shown in Table S4 & Table S5. RRT ranges are normalised with respect to the averaged median RRT at the ‘non-diseased’ location, with the median values being 1.11 and 0.43 for rest and exercise respectively (see “Supplementary data S1” for full results). Ensight 10.2.3, was used to post-process and visualise the results. *The median value for the respective metric.

In order to more clearly visualise the range of flow metrics underneath the thrombus, cases were grouped according to spatially averaged TAWSS and OSI, compared to the non-diseased reference segment. (1) More than twofold elevation of TAWSS and less than twofold elevation in OSI, compared to the reference section. (2) More than twofold elevation of TAWSS and more than twofold change in OSI. (3) Less than twofold elevation of TAWSS and more than twofold change in OSI (Fig. 2). Grouping these cases on these two flow metrics highlighted the heterogeneity in the flow metrics underlying the thrombus of culprit lesions, while identifying some overall features that are potentially important in understanding the molecular pathways that might contribute to the pathology of plaque erosion. Fifteen out of seventeen of the culprit lesions imposed an average 39.6% diameter stenosis (60.9% area stenosis). Nine of these fourteen sites of adherent thrombi showed no increase in OSI, being described most accurately by an elevation of spatially averaged TAWSS (e.g. Fig. 3, case 1), while six had elevated spatially averaged TAWSS and OSI, frequently correlating with an extension of the thrombi past the point of maximum stenosis (e.g. Fig. 3, case 10). The last two cases did not demonstrate a two-fold increase of spatially averaged TAWSS compared to reference, but showed an increase in OSI (e.g. Fig. 3, case 17) and had a lower average degree of stenosis compared to the other 15 cases.

## Discussion

Endothelial erosion of plaques mediates a substantial and possibly growing proportion of ACS^1–3, 24, 25^. The profound influence imparted by the haemodynamic environment on regulation of endothelial function^17–21^, and the response to noxious stimuli that induce endothelial dysfunction^26–28^ suggest that the haemodynamic environment is likely to influence the pathophysiology of plaque erosion. The majority of plaques imposed a mild/moderate luminal stenosis, which is in agreement with histopathology studies^7, 29, 30^. Indeed, in the absence of thrombi, a number would not have triggered intervention, highlighting the inability to identify erosion-vulnerable plaques by angiography alone. All but one of the adherent thrombi were located either proximal to, or at the point of maximum stenosis, with a subset extending beyond that point. Consequently, we identified that elevated spatially averaged TAWSS is the most common feature of the haemodynamic environment permissive for plaque erosion in human coronary arteries. Importantly, the substantial range in spatially averaged TAWSS values highlight that there is not a general threshold, which if exceeded renders the endothelium vulnerable to erosion, just that elevated flow is permissive for plaque erosion. This study makes a unique contribution to the literature as it included an estimation of the size side of branches and modelled their effect on flow and did not exclude the analysis of culprit lesions close to bifurcations. In addition, the analysis was performed on cases where mechanical removal of thrombus (by thromboaspiration, or through passage of an uninflated or partially inflated balloon catheter) was not performed prior to imaging. Mechanical removal of thrombus may favour removal of thrombus at the proximal edge of the culprit lesion. Despite these differences, the findings of this study are in complete agreement with the findings of parallel studies^31, 32^, an independent case report^33^ and one of the largest OCT study to date that analysed 209 patients with plaque erosion^11^, who observed 96% of thrombi either proximal to, or within the minimal lumen area, a region of predicted elevated flow. Taken together, this identifies that the majority of clinically significant plaque erosions occur on stenotic plaques where the endothelium is exposed to elevated flow. A recent study by Thondapu et al.^32^ demonstrated that elevated TAWSS and TAWSSG are associated with both eroded and ruptured plaques, with TAWSSG being greater at rupture sites compared to sites of erosion. This infers that elevated flow per se is insufficient to trigger endothelial erosion, but requires additional factors to induce clinically-relevant endothelial detachment.

Six of the 15 cases that displayed a greater than twofold elevation of spatially averaged TAWSS also had a greater than twofold elevation of OSI compared to the reference section; however it is possible that the thrombi extended distally to the site of erosion. In addition, the CFD simulations and subsequent analysis indicted that two cases of OCT-defined erosions occurred in regions with modest or no stenosis, where the predominant flow feature was oscillatory shear stress (defined through OSI)^34, 35^. Low time averaged wall shear stress or elevated OSI values strongly correlate with the focal predilection sites for atherosclerosis^23, 36–38^ and tend to activate endothelial cells, priming them for inflammatory activation and apoptosis^20, 39, 40^. Inducing endothelial apoptosis experimentally can initiate thrombosis^41^ supporting a role for apoptosis triggering endothelial erosion in this haemodynamic environment. An independent case report corroborates our observation that erosions can occur under conditions of oscillatory shear stress^42^.

These observations directly feed into potential mechanisms that promote plaque erosion. We have previously demonstrated that elevated flow elicits a distinct pattern of gene expression^26, 27^ and that elevated flow modifies the responses to noxious stimuli, particularly cigarette smoke extract that induces endothelial dysfunction^27, 43^. In addition, we have shown that elevated flow amplifies the Nrf2-driven antioxidant response^44^ to cigarette smoke, leading to the upregulation of oxidative stress growth inhibitor (OSGIN) 1&2, triggering endothelial detachment^43^. This links cigarette smoking, a particular risk factor for plaque erosion, with the elevated flow-dependent amplification of the Nrf2 system as a potentiating mechanism for plaque erosion. In addition, regions with high OSI favour apoptosis^40^, supporting a role for programmed cell death in endothelial erosion in this haemodynamic environment. Exposure to disturbed blood flow and engagement of TLR2 (potentially by hyaluronan fragments) stimulated endothelial apoptosis and detachment, which was enhanced by neutrophil NET formation^3, 45–47^. Therefore, elevated flow and elevated OSI may drive distinct pathways that promote clinically-relevant endothelial detachment and thrombus formation.

OCT-defined erosion requires imaging an intact fibrous cap, precluding assessment of cases with high residual thrombus burden due to the highly attenuating effect of red blood cells on the near-infrared light used by OCT. Consequently, our analysis is limited to cases where the fibrous cap could be visualised through the thrombus in each OCT frame to exclude underlying plaque rupture, and facilitate accurate delineation of the lumen profile beneath the thrombus, to obtain the pre-ACS geometry for CFD simulation. It is possible that this selection has biased the analysis of haemodynamic features of eroded plaques, however our findings are corroborated by the independent studies referenced above. Lack of patient-specific flow measurements, required the use of previously published artery-specific flow rates for the simulation. The movement of the coronary arteries throughout the cardiac cycle was not included in the model; however, it is not anticipated that the elevated and oscillatory flow metrics associated with the stenotic plaques would not be severely affected by arterial movement. We analysed and averaged the entire area under the thrombus, which might extend distal to the denuded area, as there is no way of defining area of endothelial loss by OCT, this may also increase observation of increased OSI in the measurements, as the thrombus sometimes extended beyond the point of maximum stenosis where OSI is prevalent. Currently, the reconstruction process is very resource intensive, limiting the number of arteries for study. Automation of the methodology may allow higher-throughput analysis of flow environment and application to datasets of enhanced size. For this reason, we did not study non-culprit lesions, patients with stable coronary artery disease, or cases of plaque rupture to identify if there were any flow characteristics that were unique to plaque erosion cases, which would have enhanced this analysis.

These findings suggest that the majority of OCT-defined erosions occur on moderately stenotic plaques in regions of elevated flow; however, a small number also occur in the absence of an elevation of flow, with high oscillatory flow. While the average metrics indicate that elevated flow and flow gradient significantly increase in the area covered by the thrombus, the spread of the data indicate there is no definitive flow threshold that induces plaque erosion. It is also likely that these particular flow metrics exist on other stenotic plaques that have not experienced clinically relevant plaque erosion. Therefore, despite the haemodynamic environment profoundly influencing endothelial function, elevated or oscillatory flow per se is unlikely to be causal for plaque erosion and might better be described as permissive, amplifying the effects of smoking or other as yet unidentified triggers of plaque erosion.

## Materials and methods

In the present work, we focus on linking blood flow induced shear stress metrics to the location of plaque erosion. Determination of patient-specific shear stress maps were generated using computational fluid dynamics (CFD) to generate patient-specific wall shear stress maps. Application of this tool requires in-depth knowledge of the underlying engineering principles, and to ensure appropriate application of CFD, we followed the recommendations of an expert consensus group^48^. Details regarding the 3D reconstruction and numerical procedure are given in “Supplementary material S1”. All methods were carried out in accordance to relevant guidelines and regulations. Fully anonymised patient data obtained during routine clinical treatment was retrospectively examined in this study. Ethical approval for the use of this anonymised data was approved by local hospital board review (Policlinico A. Gemelli & University Hospitals Bristol). Specific informed consent was waived by internal review boards (Policlinico A. Gemelli & University Hospitals Bristol). This study falls outside the scope of the UK policy framework for health and social care research and was registered with University Hospitals Bristol and Weston NHS Foundation Trust as a service evaluation. It is an analysis of routinely collected anonymized data, and followed the national “Guidance on the use of patient images obtained as part of standard care for teaching, training and research” issued by the Royal College of Radiologists, UK.

### Patient demographics

Twenty cases were obtained from a database of OCT-defined erosions from Bristol and Italian retrospective datasets, where three reviewers agreed that the intact fibrous cap could be visualised underneath the thrombus in every frame, excluding the possibility of plaque rupture and allowing the pre-PCI geometry to be extracted. Thromboaspiration was not performed prior to OCT imaging, minimising the risk of iatrogenic disruption of vessel geometry and lumen wall. From these 20 cases, three cases were subsequently excluded because of data quality, with full reconstruction of the coronary artery luminal architecture from 17 patients (LAD, n = 7; LCX, n = 5; RCA, n = 5). Demographics are presented in Table 1.

**Table 1:** Patient demographics.

	Case	Age	Sex	Diagnosis	Vessel	Smoking status	Underlying plaque
Bristol	1	53	Male	STEMI	RCA	Never	ThCFA
	2	51	Male	STEMI	LAD	Current	ThCFA
	3	28	Male	NSTEACS	LAD	Prior	ThCFA
	4	48	Female	NSTEACS	LAD	Current	ThCFA
	5	37	Female	STEMI	LAD	Prior	ThCFA
	6	28	Male	STEMI	LAD	Never	ThCFA
	7	59	Male	STEMI	LMS	Prior	ThCFA
	**8**	**52**	**Male**	**NSTEACS**	**Cx**	**Never**	**ThCFA**
	9	60	Female	NSTEACS	LAD	Prior	ThCFA
	10	64	Male	STEMI	Cx	Never	Calcified ThCFA
	**11**	**20**	**Female**	**STEMI**	**LAD**	**Current**	**ThCFA**
	12	44	Male	NSTEACS	LAD	Current	Calcified ThCFA
	13	39	Male	NSTEACS	LAD	Current	Calcified ThCFA
Rome	14	55	Male	NSTEACS	Cx	Current	ThCFA
	15	63	Male	STEMI	LAD	Never	Calcified ThCFA
	**16**	**71**	**Female**	**STEMI**	**LAD**	**Never**	**Calcified ThCFA**
	17	67	Male	NSTEACS	Cx	Current	ThCFA
	18	41	Male	NSTEACS	RCA	Current	Calcified ThCFA
	19	53	Male	NSTEACS	LAD	Current	ThCFA
	20	49	Male	NSTEACS	RCA	Never	ThCFA

Cases in bold were excluded due to image quality during geometry reconstruction as described below.

*STEMI* ST-elevated myocardial infarction, *NSTEACS* non-ST elevated acute coronary syndrome, *RCA* right coronary artery, *LAD* left anterior descending artery, *Cx* circumflex artery, *LMS* left main stem coronary artery, *ThCFA* thin capped fibroatheroma.

### Arterial geometry reconstruction

**Figure 4:**
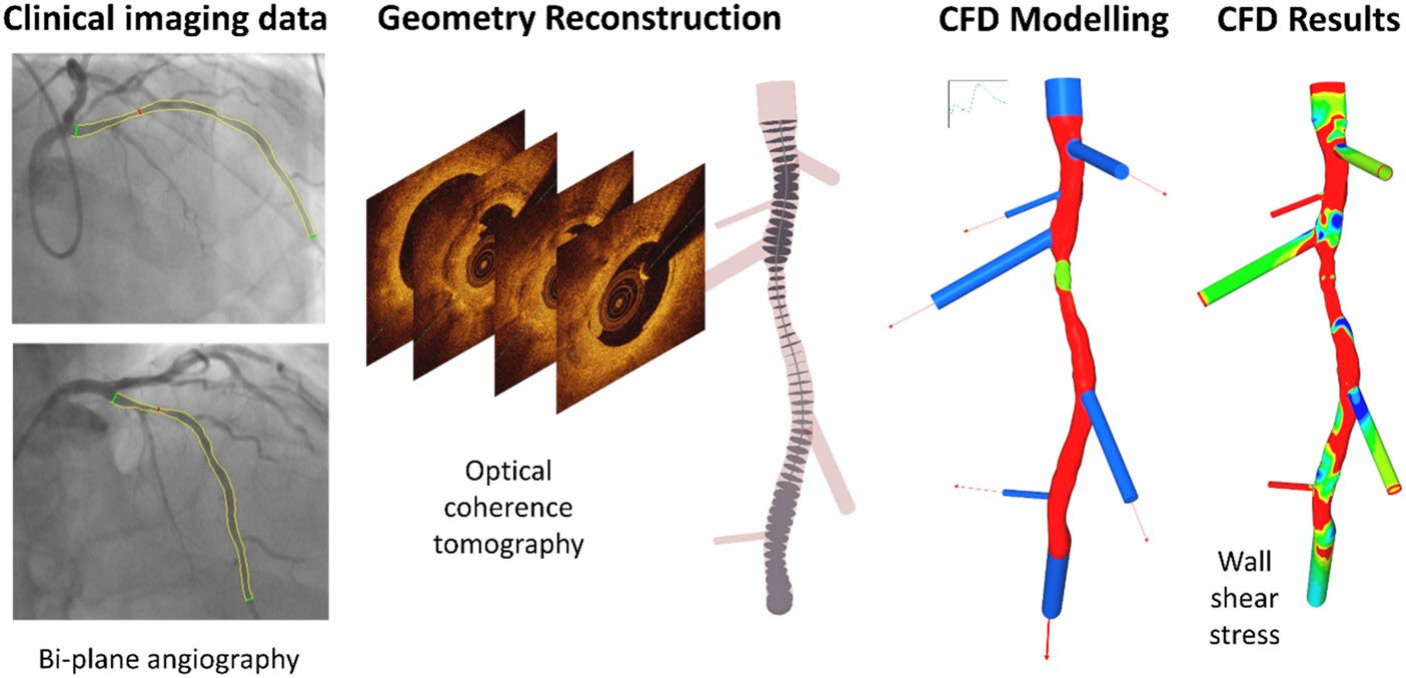
Probing the haemodynamic conditions permissive for plaque erosion. OCT and bi-plane angiography of coronary arteries were collected and used to reconstruct lumen geometries (n = 17). Red sections are high accuracy reconstructions from hybrid OCT/bi-plane angiography, blue sections use bi-plane angiography and OCT to determine the diameter and branch of angle for the flow extensions, with adherent thrombus in green. At ‘rest’ and ‘exercise’ pulsatile flow conditions were simulated for 4 cardiac cycles. Haemodynamic flow results were post-processed to quantify additional wall shear-based haemodynamic metrics of interest. Ensight 10.2.3, was used to post-process and visualise the results.

To gain insights into mechanisms responsible for endothelial erosion from plaques, we first sought to define the luminal geometry to compute the local haemodynamic environment at the site of OCT-defined erosions in ACS patients. The lumen geometries of LAD (left anterior descending artery), LCX (left circumflex artery) and RCA (right coronary artery) arteries were reconstructed by combining geometrical information derived from bi-plane cineangiography and the high-definition lumen profile derived from OCT. The luminal contour was identified beneath the thrombus, establishing the structure prior to the ACS event^49^. The lumen surface with adherent thrombi was mapped onto the combined arterial geometry and assumed to be synonymous with the area of endothelial erosion (*Fig. **4*).

### Computational fluid dynamics

The 3D geometry was discretized to generate a finite element mesh ANSYS-Meshing (Version 19.0). A mesh refinement study was conducted to ensure that the computed wall shear stress was independent of the element size. The CFD solver ANSYS-CFX (Version 19.0) was used for the blood flow simulations. Numerical assessment of haemodynamic environments was conducted under both resting and stress conditions using simulations of artery-specific waveforms.

Blood was defined as a Newtonian and incompressible fluid with dynamic viscosity of 0.004 Pa s^−1^ and density of 1060 kg m^−3^^50^. Blood flow through stenosed arteries under exercise conditions can potentially develop turbulence^51^, therefore, k–ω shear stress transport model^52^ was employed as the turbulence model in these simulations as it is considered the best suited turbulence model for capturing the turbulent transition in coronary arteries^51, 53, 54^. For all cases, no-slip boundary conditions were applied to all walls^55^. Time-dependent coronary velocity profiles were prescribed at the inlets based on LAD, LCX and RCA flow data adapted from Kim et al.^56^, with a rigid wall model^57^. At all the outlets, except for the most distal, an outlet velocity was prescribed using scaled versions of the inlet profile to satisfy Doriot’s fit^55, 58^. Simulations were run for four cardiac cycles (4 s) with a time step size of 1.25 ms and results were recorded during the final cardiac cycle.

### Haemodynamic metrics and analyses

The commercial visualisation tool, Ensight 10.2.3, was used to post-process the results and extract widely used wall shear-based haemodynamic metrics, specifically; Time-Averaged Wall Shear Stress (TAWSS)^59, 60^, maximum wall shear stress (WSS_max_—maximum area-weighted average wall shear stress over time, evaluated at the thrombus), Oscillatory Shear Index (OSI)^34, 60^, Relative Residence Time (RRT)^34, 59, 61^ and Time-Averaged Wall Shear Stress Gradient (TAWSSG)^34, 62^ according to Eqs. (1) – (4).

1 $$TAWSS= \frac{1}{T}{\int }_{0}^{T}\left|{\overrightarrow{\tau }}_{w}\right|dt$$

2 $$OSI= \frac{1}{2}\left(1-\frac{\left|{\int }_{0}^{T}{\overrightarrow{\tau }}_{w}dt\right|}{{\int }_{0}^{T}\left|{\overrightarrow{\tau }}_{w}\right|dt}\right)$$

3 $$RRT=\frac{1}{\frac{1}{T}\left|{\int }_{0}^{T}{\overrightarrow{\tau }}_{w}dt\right|}$$

4 $$TAWSSG=\frac{1}{T}{\int }_{0}^{T}\sqrt{{\left(\frac{\partial {\tau }_{x}}{\partial x}\right)}^{2}+{\left(\frac{\partial {\tau }_{y}}{\partial y}\right)}^{2}+{\left(\frac{\partial {\tau }_{z}}{\partial z}\right)}^{2}} dt$$

In the above equations, ${\overrightarrow{\tau }}_{w}$ is the WSS vector and *T* is the time period of the flow cycle.

A non-diseased reference segment of approximately 1 cm in length was identified adjacent to the culprit lesion. Where possible, this was just proximal to the culprit lesion, unless there was insufficient disease-free artery proximal to the culprit lesion, in which case the closest disease-free distal section was used. Differences in flow metrics between the reference segment and entire area with adherent thrombi were assessed by paired T-test.

## Supplementary Information

Supplementary Information.

## Abbreviations

OCT: Optical coherence tomography
CFD: Computational fluid dynamic
ACS: Acute coronary syndromes
LAD: Left anterior descending artery
LCX: Left circumflex artery
RCA: Right coronary artery
TAWSS: Time averaged wall shear stress
WSSmax: Maximum wall shear stress
TAWSSG: Time averaged wall shear stress gradient
RRT: Relative residence time
OSI: Oscillatory shear index
