# Supplementary material for: Identification of the haemodynamic environment permissive for plaque erosion
Source: Sci Rep. 2021 Mar 31;11:7253. doi: 10.1038/s41598-021-86501-x (PMC8012657; doi:10.1038/s41598-021-86501-x)
Supplement: Supplementary file 1 — Supplementary Information. [file 41598_2021_86501_MOESM1_ESM.docx]

**Identification of The Haemodynamic Environment Permissive for Plaque Erosion**

Michael McElroy^1^, Yongcheol Kim^2^, Giampaolo Niccoli^3^, Rocco Vergallo^3^, Alexander Langford-Smith^4^, Filippo Crea^3^, Frank Gijsen^5^, Thomas Johnson^7*^, Amir Keshmiri^1*^ and Stephen J White^4*†^

**Supplementary information**

**Methods**

**A1. Lumen geometry reconstruction**

The geometry was constructed by systematically going through the following 5 stages.

1. **3D centreline extraction.** The medical imaging software QAngio XA 3D by Medis (Leiden, Netherlands) was used to export the bi-plane angiography imaging. The geometry was then imported into Vascular Modelling Toolkit (VMTK) where the Cartesian co-ordinates of the artery centreline were extracted.
2. **OCT images segmentation.** The Cartesian co-ordinates of the OCT frames were exported from QCU-CMS by Medis (Leiden, Netherlands). This data was then processed and used to build geometrical representations of each frame in SOLIDWORKS. The luminal contour of each frame was smoothed to eliminate artefact or non-uniform rotational deformation present in the OCT data.
3. **Registration.** Manually interpreting the bi-plane angiography images enabled side branch centreline positions to be defined and built into the geometrical model. OCT frames were then stacked perpendicularly with the lumen centroid of each frame intersecting the 3D centreline(61-63). The distance between the OCT frames was defined by the catheter pullback speed(64). This stacking procedure is similar to the ANGUS technique used in similar studies in the literature(64-67). For the stenosis region, the sampling rate was approximately 1 to 2, similar to methods used in the literature(63). Higher sampling rate had to be applied where there was no stenosis. OCT frames at branch bifurcation sites were then rotationally orientated by aligning landmarks on the OCT frames that indicate a bifurcation site with the peripheral branches. The interim frames were then orientated using an interpolation technique. Both the registration and subsequent processes were conducted using SOLIDWORKS.
4. **Surfacing.** A lofting technique was then used between each OCT frame sequentially to create the final lumen surface. Peripheral branch diameters were extracted from the relative OCT frames and the branch walls were extruded in the model with fillets of 0.25 mm in radius applied at the bifurcation sites. Flow extensions were affixed to the inlet (one diameter in length) and outlets (seven diameters in length) to ensure physiologically accurate flow profiles within the artery(66,68,69)*.* Inlet and outlet diameters for each case are shown in *Table S1*.
5. **Adhered thrombus identification.** The adhered thrombus sites in each OCT frame was noted sequentially and transferred onto the lumen surface. This was used to define where the adhered thrombus has formed on the lumen wall and was later used to extract haemodynamic metrics associated with atherosclerotic plaque erosion at this site. For information regarding the severity of stenosis for each case, refer to *Table S2*.

**A2. Computational Domain**

In order to ensure the accuracy of the simulations, a series of 10 steady-state computations with various mesh refinement levels (ranging from 0.7 to 14.5 million elements) were conducted to test mesh convergence (see Figure S1). Case 9 was used for the mesh convergency study as it was deemed to represent the general population of the cases studied by including common characteristics, such as the adhered thrombus site being in close proximity to a stenosis, having a relatively large number of outlets and the stenosis was a sufficient distance distal from the inlet to allow for flow to properly develop. The geometries were meshed using ANSYS-Meshing (Version 19.0). The mesh was based on a finite volume hybrid mesh consisting of tetrahedral elements within the core region and prism layers (3 elements thick) near the wall to allow for large spatial velocity gradients. Velocity representing flow of 5 cc/s was applied to the inlet based on the maximum velocity from the pulsatile waveform of the LCX artery of a 36 year old male subject during light exercise (32) (as shown in Figure S3). Flow ratios are then applied to the outlets, except for the most distal, which had a zero pressure condition applied, details are given in Table S3. The velocity within the stenosis, as well as the overall volume were monitored. The maximum change in velocity was 0.5 % from 5.5 to 6.3 million elements. Velocity iso-surfaces as well as wall shear stress contours were qualitatively assessed. The mesh settings used in the 6.3 million elements study were deemed adequate for this application, and was therefore used for all subsequent simulations. The final mesh chosen for Case 9 is shown in Figure S2. It is worth noting that the mesh densities used in this work are approximately triple that used in a similar study by Timmins *et al.*(69), which further supports the confidence in the mesh settings used.

**A3. Boundary conditions**

At all the outlets, except for the most distal, an outlet velocity was prescribed using scaled versions of the inlet profile to satisfy Doriot’s fit (31,34) (Eq. 1).

| $\dot{m}=d^{2.27}$ | (1) |
| --- | --- |

where $\dot{m}$ is the mass flow rate and *d* is the vessel diameter.

At the remaining, most distal, outflow tract, a traction free boundary condition was applied. This method is an accurate way of prescribing outlet conditions within coronary arteries (31,34). The velocities at the inlet and outlets were assigned as a velocity normal to the boundary. The boundary conditions for each case are summarised in Table S3.

*Table* *S1 – Inlet & outlet diameters of the coronary artery lumen geometries used in this study. Outlet numbers ascend in order as they become more distal from the inlet.*

|  | Diameter (mm) | | | | | | | |
| --- | --- | --- | --- | --- | --- | --- | --- | --- |
|  | Inlet | Outlet 1 | Outlet 2 | Outlet 3 | Outlet 4 | Outlet 5 | Outlet 6 | Outlet 7 |
| Case 1 | 3.48 | 1.6 | 1 | 1.6 | 0.2 | 2.3 | - | - |
| Case 2 | 2.76 | 0.6 | 0.9 | 0.8 | 0.4 | 1.1 | 1.3 | 2.05 |
| Case 3 | 2.24 | 2.2 | 0.9 | 0.9 | 1.5 | 2.87 | - | - |
| Case 4 | 2.41 | 0.8 | 1 | 0.7 | 1.61 | - | - | - |
| Case 5 | 3.66 | 2 | 0.5 | 0.8 | 0.7 | 2.91 | - | - |
| Case 6 | 4.55 | 2.3 | 0.4 | 0.2 | 1.7 | 1.6 | 0.9 | 2.77 |
| Case 7 | 3.9 | 1.7 | 1.5 | 0.6 | 1.2 | 0.7 | 3.1 | - |
| Case 8 | 3.66 | 1 | 1 | 0.5 | 2.7 | - | - | - |
| Case 9 | 3.79 | 1.6 | 0.7 | 1.8 | 1.6 | 0.8 | 2.79 | - |
| Case 10 | 3.71 | 0.7 | 0.8 | 0.3 | 1 | 2.6 | - | - |
| Case 11 | 3.34 | 1.2 | 0.9 | 1.9 | 1.6 | 3.02 | - | - |
| Case 12 | 4.04 | 1.7 | 1 | 1.5 | 2.46 | - | - | - |
| Case 13 | 3.33 | 0.6 | 0.7 | 1.2 | 1.1 | 2.26 | - | - |
| Case 14 | 4.75 | 3.8 | 2.1 | 1.9 | 2.4 | 2.89 | - | - |
| Case 15 | 3.32 | 1.4 | 0.6 | 0.8 | 1.4 | 1.67 | - | - |
| Case 16 | 3.31 | 1.6 | 1.3 | 1.7 | 0.7 | 2.36 | - | - |
| Case 17 | 3.67 | 1.6 | 1.4 | 1.6 | 0.7 | 2.61 | - | - |

*Table S2 – Average diameter of areas of interest and the percentage of the stenosis.*

|  | Non-diseased | Under thrombus | Percentage Stenosis | |
| --- | --- | --- | --- | --- |
|  |  |  |  |  |
|  | Average diameter (mm) | | Diameter (%) | Area (%) |
| Case 1 | 2.9 | 1.4 | 51.9 | 76.9 |
| Case 2 | 2.9 | 2.3 | 20.7 | 37.2 |
| Case 3 | 3.1 | 2.1 | 32.4 | 54.2 |
| Case 4 | 2.8 | 2.0 | 28.2 | 48.4 |
| Case 5 | 3.3 | 2.3 | 30.0 | 51.0 |
| Case 6 | 3.6 | 2.5 | 29.0 | 49.6 |
| Case 7 | 3.9 | 1.3 | 66.2 | 88.6 |
| Case 8 | 3.7 | 1.4 | 62.3 | 85.8 |
| Case 9 | 2.3 | 1.6 | 30.9 | 52.3 |
| Case 10 | 3.7 | 1.4 | 61.6 | 85.3 |
| Case 11 | 3.3 | 2.3 | 30.9 | 52.3 |
| Case 12 | 3.2 | 1.9 | 41.1 | 65.3 |
| Case 13 | 2.7 | 1.2 | 57.4 | 81.9 |
| Case 14 | 2.9 | 2.6 | 9.00 | 17.2 |
| Case 15 | 2.5 | 1.4 | 43.3 | 67.8 |
| Case 16 | 2.6 | 2.2 | 16.3 | 30.0 |
| Case 17 | 2.7 | 3.6 | -32.8 | -76.5 |
| Minimum | 2.3 | 1.2 | -32.8 | -76.5 |
| Quartile 1 | 2.7 | 1.4 | 28.2 | 48.4 |
| Median | 2.9 | 2.0 | 30.9 | 52.3 |
| Quartile 3 | 3.3 | 2.3 | 51.9 | 76.9 |
| Maximum | 3.9 | 3.6 | 66.2 | 88.6 |

*Table S3 – Summary of boundary conditions applied for each case. For all the outlets except the most distal, the outflow ratio was calculated according to Doriot’s fit (Eq. 1).*

|  | Boundary condition | | | | | | | |
| --- | --- | --- | --- | --- | --- | --- | --- | --- |
|  | Inlet | Outlet 1 | Outlet 2 | Outlet 3 | Outlet 4 | Outlet 5 | Outlet 6 | Outlet 7 |
| Case 1 | $\dot{m}_{RCA}(t)$ | 21.60 % | 7.43 % | 21.60 % | 0.19 % | 0 Pa | - | - |
| Case 2 | $\dot{m}_{LAD}(t)$ | 3.14 % | 7.88 % | 6.03 % | 1.25 % | 12.43 % | 18.16 % | 0 Pa |
| Case 3 | $\dot{m}_{LAD}(t)$ | 28.46 % | 3.74 % | 3.74 % | 11.93 % | 0 Pa | - | - |
| Case 4 | $\dot{m}_{LAD}(t)$ | 12.08 % | 20.04 % | 8.92 % | 0 Pa | - | - | - |
| Case 5 | $\dot{m}_{RCA}(t)$ | 27.75 % | 1.19 % | 3.47 % | 2.56 % | 0 Pa | - | - |
| Case 6 | $\dot{m}_{LAD}(t)$ | 27.70 % | 0.52 % | 0.11 % | 13.95 % | 12.15 % | 3.29 % | 0 Pa |
| Case 7 | $\dot{m}_{RCA}(t)$ | 15.76 % | 11.86 % | 1.48 % | 7.15 % | 2.10 % | 0 Pa | - |
| Case 8 | $\dot{m}_{LCX}(t)$ | 8.52 % | 8.52 % | 1.77 % | 0 Pa | - | - | - |
| Case 9 | $\dot{m}_{LCX}(t)$ | 13.88 % | 2.12 % | 18.13 % | 13.88 % | 2.88 % | 0 Pa | - |
| Case 10 | $\dot{m}_{LCX}(t)$ | 4.10 % | 5.55 % | 0.60 % | 9.21 % | 0 Pa | - | - |
| Case 11 | $\dot{m}_{RCA}(t)$ | 6.94 % | 3.61 % | 19.70 % | 13.33 % | 0 Pa | - | - |
| Case 12 | $\dot{m}_{LAD}(t)$ | 22.86 % | 6.85 % | 17.21 % | 0 Pa | - | - | - |
| Case 13 | $\dot{m}_{LCX}(t)$ | 3.17 % | 4.50 % | 15.28 % | 12.54 % | 0 Pa | - | - |
| Case 14 | $\dot{m}_{LAD}(t)$ | 42.46 % | 11.05 % | 8.80 % | 14.96 % | 0 Pa | - | - |
| Case 15 | $\dot{m}_{LCX}(t)$ | 25.48 % | 3.72 % | 7.15 % | 25.48 % | 0 Pa | - | - |
| Case 16 | $\dot{m}_{LAD}(t)$ | 18.70 % | 11.67 % | 21.46 % | 2.86 % | 0 Pa | - | - |
| Case 17 | $\dot{m}_{RCA}(t)$ | 16.87 % | 12.46 % | 16.87 % | 2.58 % | 0 Pa | - | - |
| $\dot{m}_{LAD}(t)$, $\dot{m}_{LCX}(t)$ and $\dot{m}_{RCA}\left( t \right)$ represent time-dependent mass flow rates of LAD, LCX and RCA arteries respectively. Percentage values represent outflow ratios. The most distal outlet has a zero pressure condition. Outlet numbers ascend in order as they become more distal from the inlet | | | | | | | | |

| 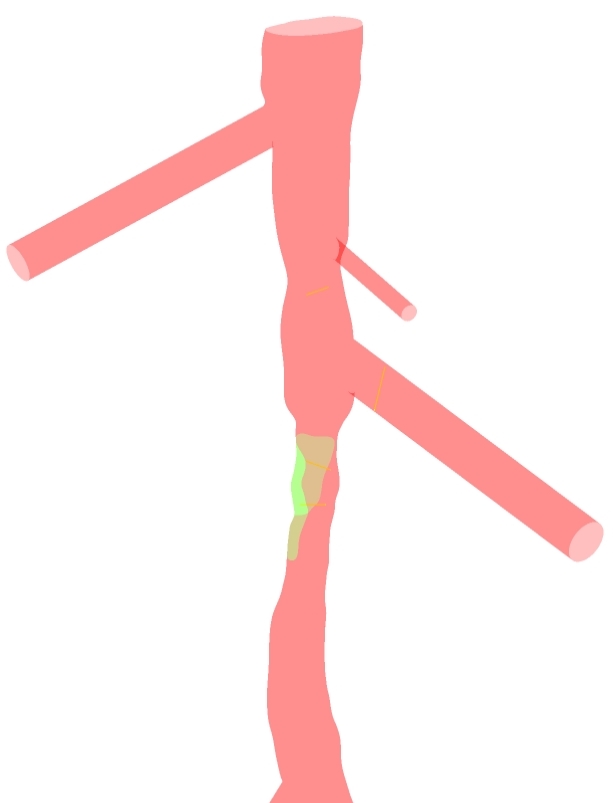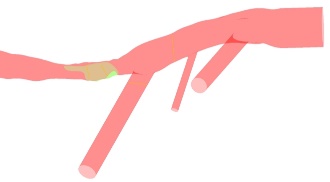  L1  L2  L3  L4  L3  L2  L1  Volume  L4 |
| --- |

*Figure S1 – Mesh convergence history plots. Velocity was monitored at 4 monitoring line locations, as well as the maximum volume velocity. Two lines (L1 & L2) are located within the stenosis to monitor the flow at the region of greatest interest. Monitoring line (L3) was placed within the branch proximal and in close proximity to the stenosis and adhered thrombus. Monitoring line (L4) is placed proximal to the aforementioned branch and within the LCX. These monitoring locations were chosen as they would capture the changes in the flow environment within the areas of most interest.*

| 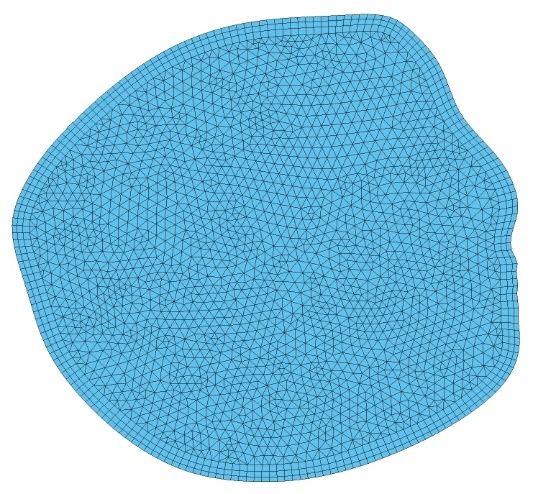  (a) | 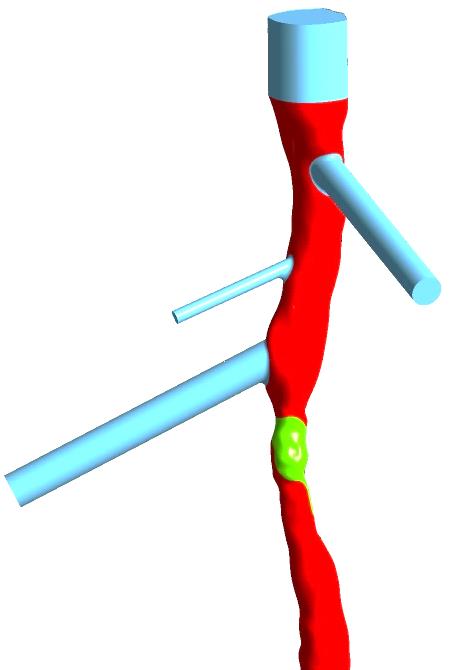  (b)  (c)  (a) |
| --- | --- |
| 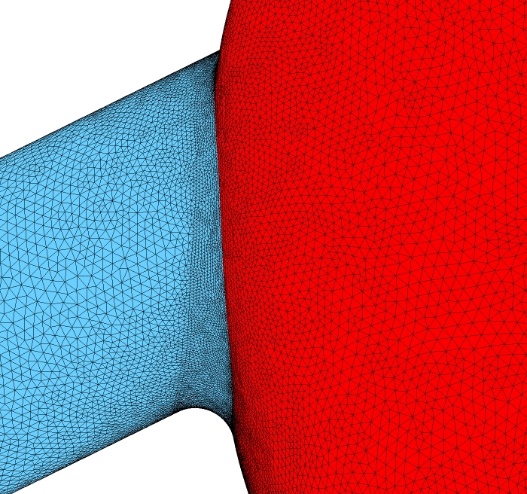  (b) | 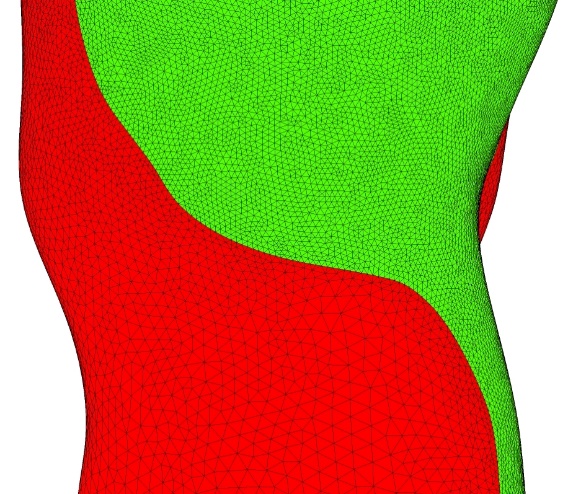  (c) |

*Figure S2 – Example of the chosen mesh setting used for all simulations. This mesh represents the optimal performing mesh taken from the mesh independency study (Case 9), in this case, the mesh has 6.3 million elements. (a) Cross-sectional view at the inlet, here the prism layers are visible on the lumen walls. (b) & (c) Refined face sizing (0.02 mm) at the bifurcation and adhered thrombus regions. Note: Different colours in (b) and (c) represent regions with different mesh refinement requirements but there is no discontinuity in the whole computational domain.* *Ensight 10.2.3, was used to post-process and visualise the results*

| 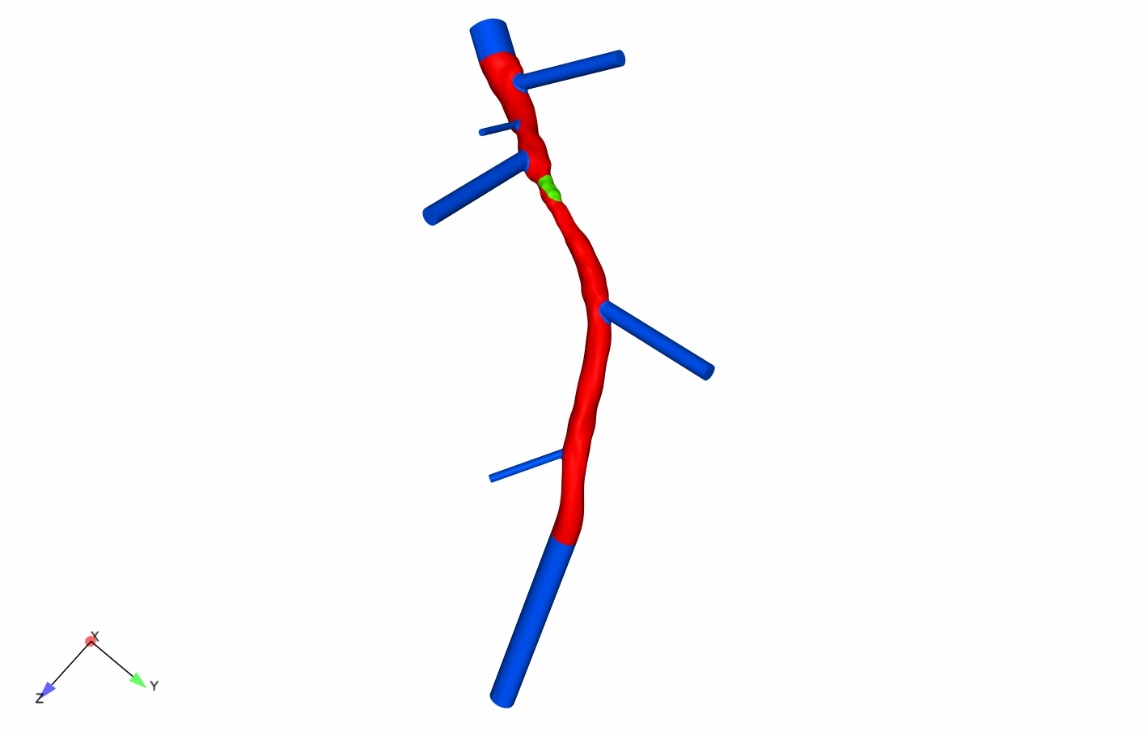  2.88 %  2.12 % %  Exercise  Rest  13.88 %  13.88 %  18.13 %  0 Pa |  |
| --- | --- |
|  | LCX |
|  | LAD |
|  | RCA |

*Figure S3 – Boundary conditions applied at the inlet and outlets. The LAD, LCX and RCA velocity profiles adapted from Kim et al. (32) and used for the simulations. These profiles were from a patient at both rest and light exercise. Case 9 (LCX) is used here as an example.* *Ensight 10.2.3, was used to post-process and visualise the results*

**Supplementary Data**

* The median value for the respective metric.

|  |  | (a) | (b) | | | | | | | |
| --- | --- | --- | --- | --- | --- | --- | --- | --- | --- | --- |
|  |  | Geometry | TAWSS (Pa) | | OSI (-) | | RRT (-) | | TAWSSG (Pa/mm) | |
|  |  |  | Rest | Exercise | Rest | Exercise | Rest | Exercise | Rest | Exercise |
|  |  |  | 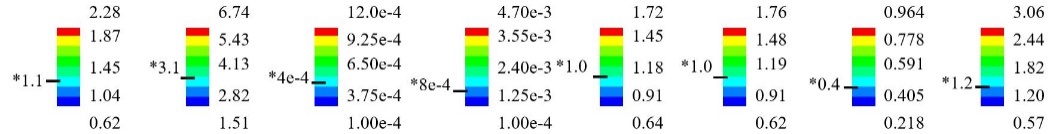 | | | | | | | |
|  |  |  |  |  |  |  |  |  |  |  |
|  |  |  |  |  |  |  |  |  |  |  |
|  |  |  |  |  |  |  |  |  |  |  |
|  |  |  |  |  |  |  |  |  |  |  |
| Case 1 |  | 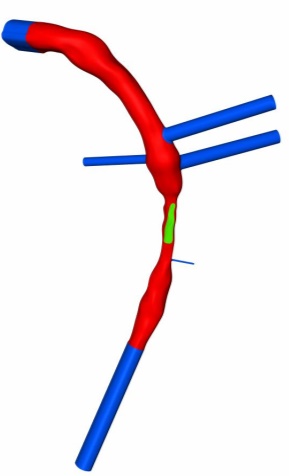 | 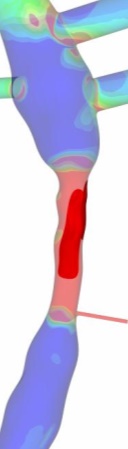 | 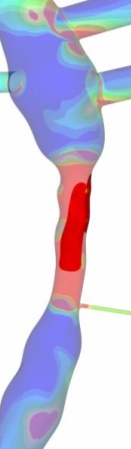 | 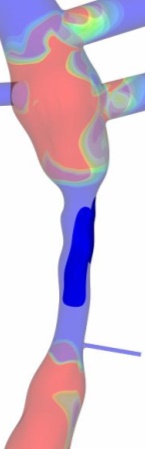 | 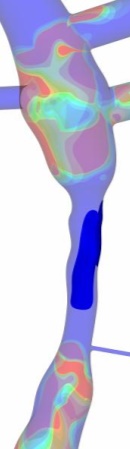 | 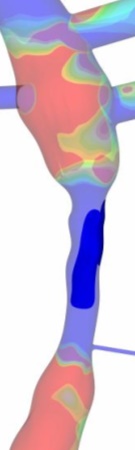 | 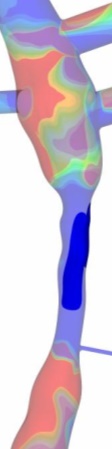 | 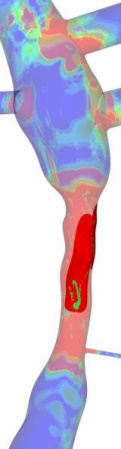 | 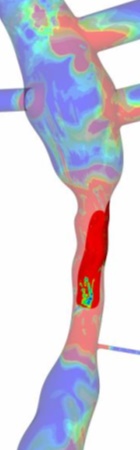 |
| Case 2 |  | 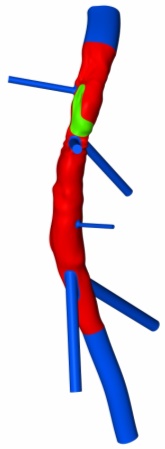 | 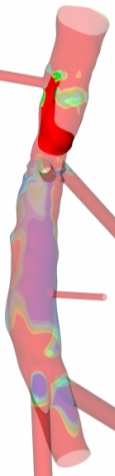 | 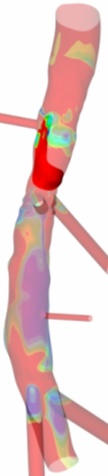 | 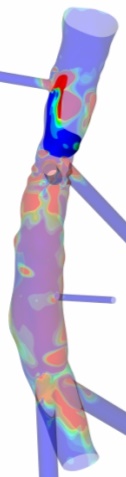 | 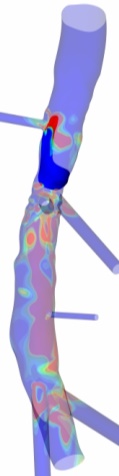 | 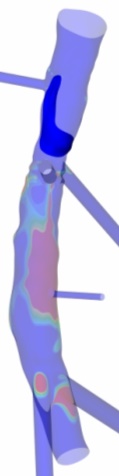 | 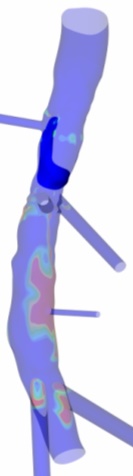 | 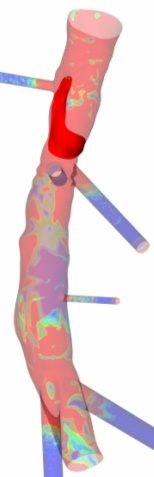 | 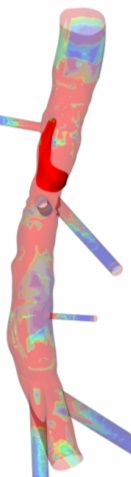 |
| Case 3 |  | 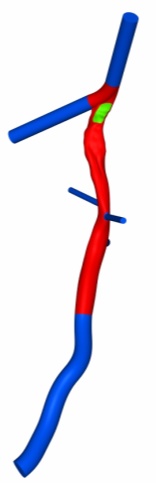 | 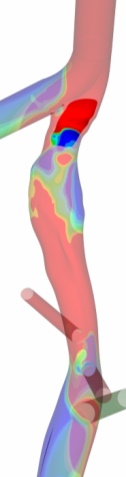 | 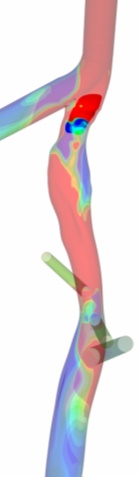 | 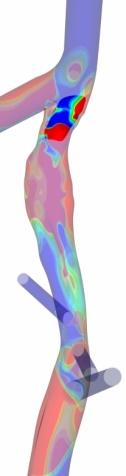 | 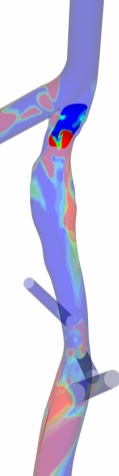 | 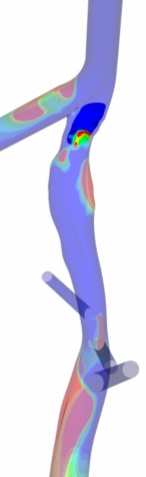 | 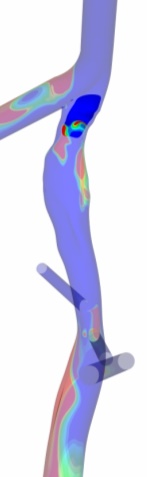 | 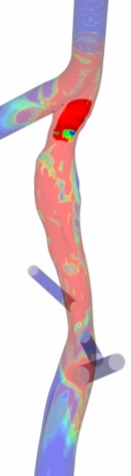 | 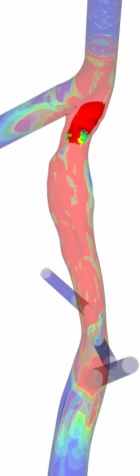 |
| Case 4 |  | 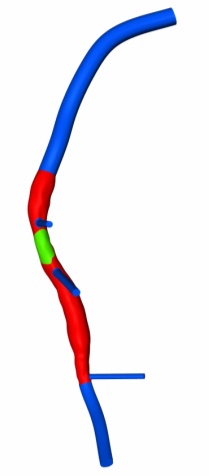 | 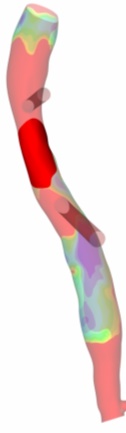 | 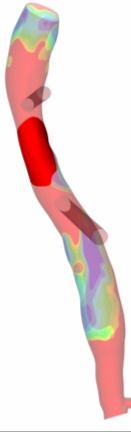 | 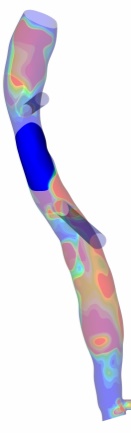 | 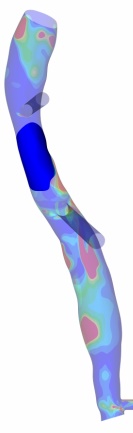 | 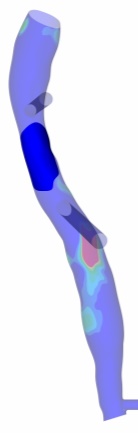 | 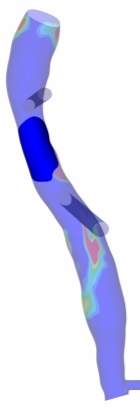 | 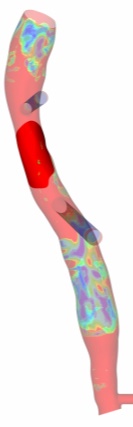 | 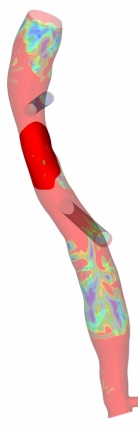 |
| Case 5 |  | 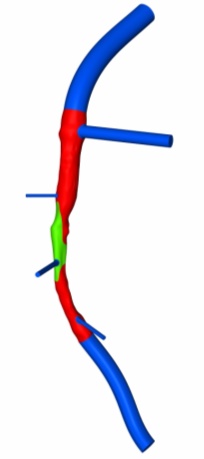 | 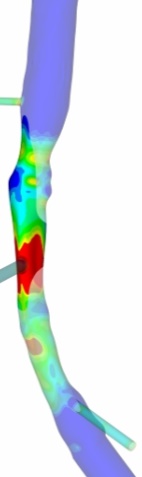 | 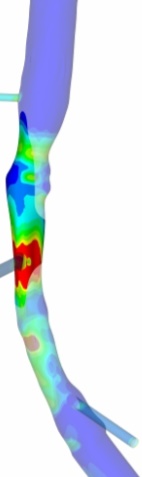 | 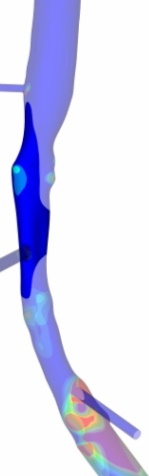 | 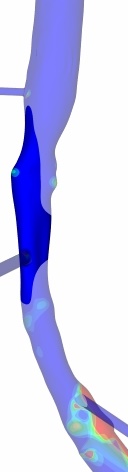 | 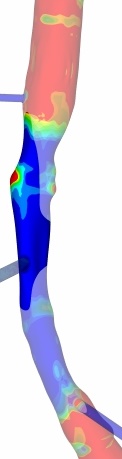 | 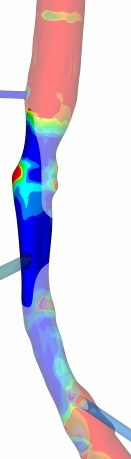 | 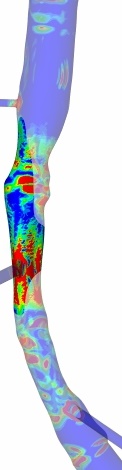 | 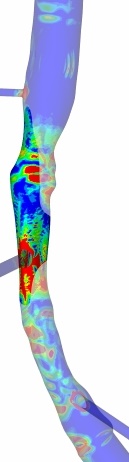 |
| Case 6 |  | 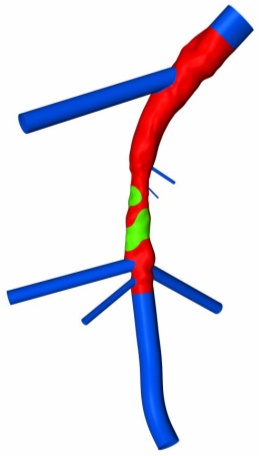 | 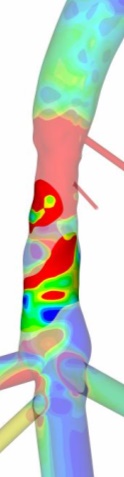 | 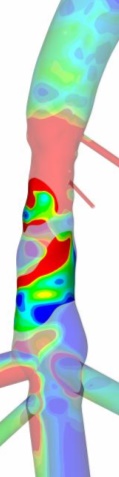 | 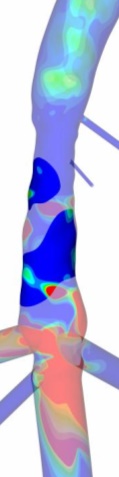 | 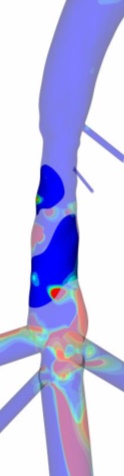 | 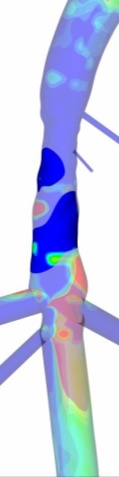 | 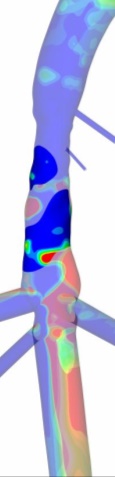 | 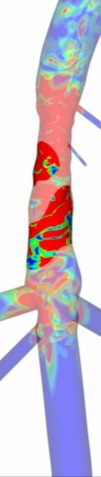 | 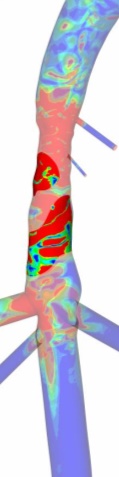 |
| Case 7 |  | 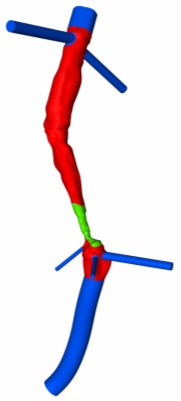 | 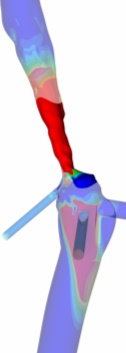 | 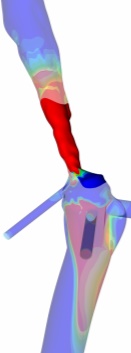 | 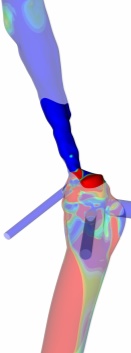 | 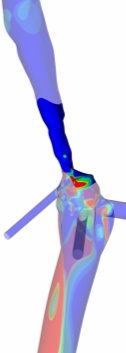 | 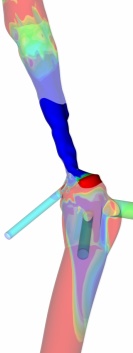 | 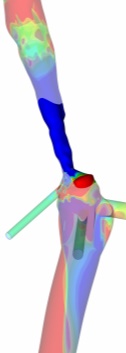 | 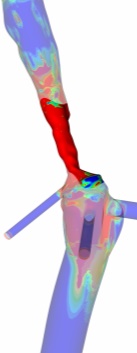 | 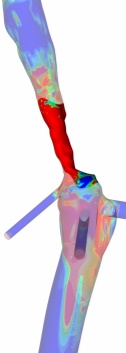 |
| Case 8 |  | 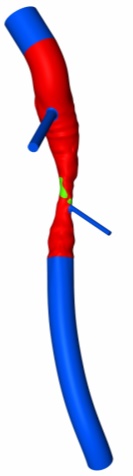 | 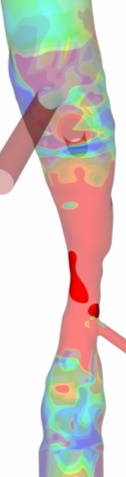 | 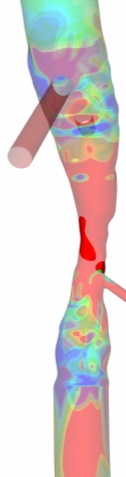 | 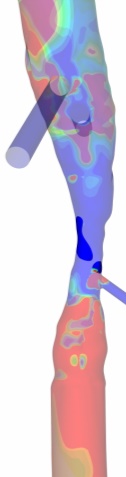 | 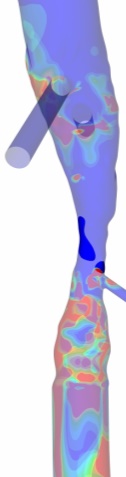 | 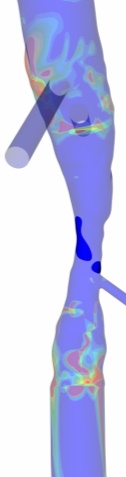 | 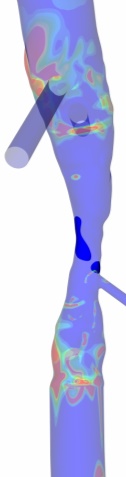 | 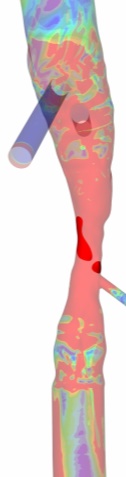 | 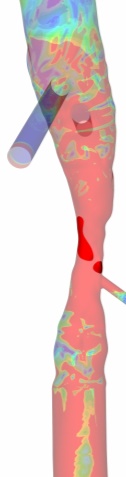 |
| Case 9 |  | 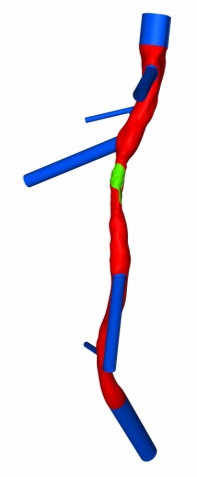 | 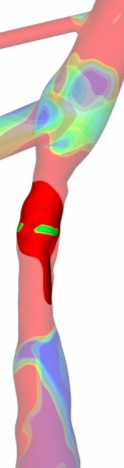 | 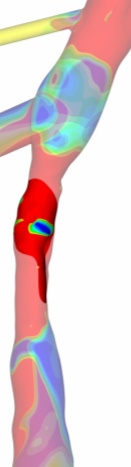 | 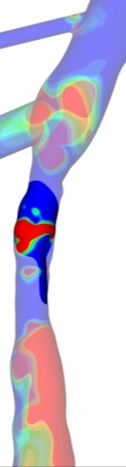 | 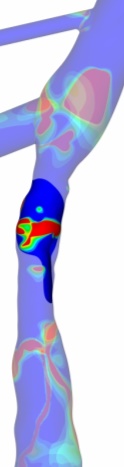 | 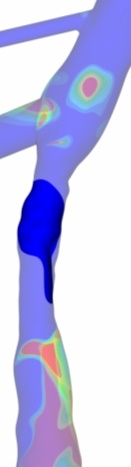 | 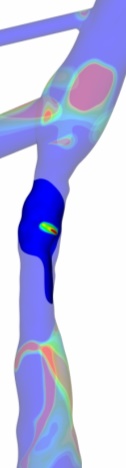 | 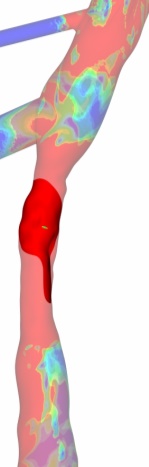 | 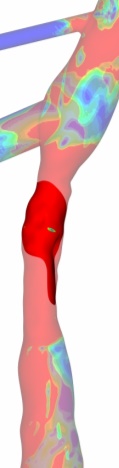 |
| Case 10 |  | 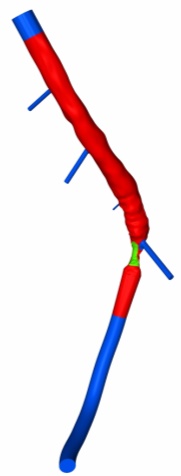 | 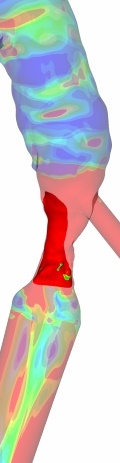 | 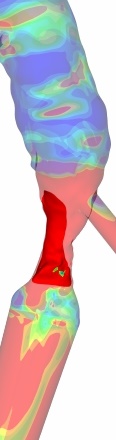 | 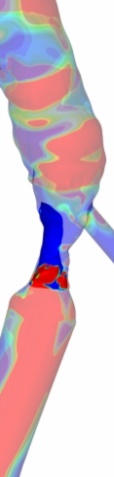 | 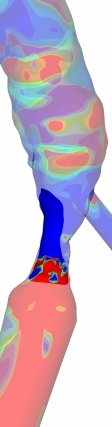 | 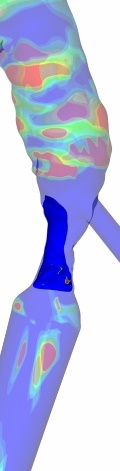 | 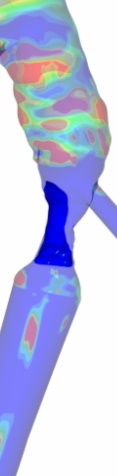 | 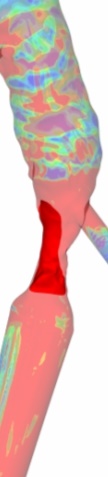 | 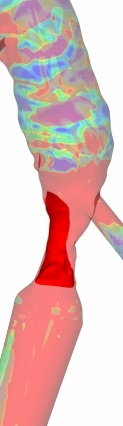 |
| Case 11 |  | 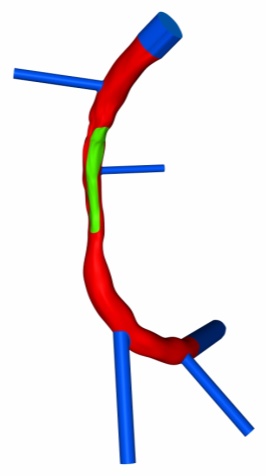 | 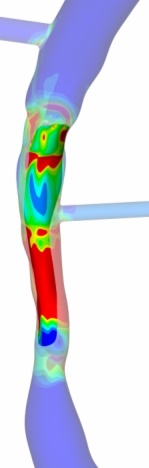 |  |  |  |  |  |  |  |
| Case 12 |  |  |  |  |  |  |  |  |  |  |
| Case 13 |  |  |  |  |  |  |  |  |  |  |
| Case 14 |  |  |  |  |  |  |  |  |  |  |
| Case 15 |  |  |  |  |  |  |  |  |  |  |
| Case 16 |  |  |  |  |  |  |  |  |  |  |
| Case 17 |  |  |  |  |  |  |  |  |  |  |

Figure S4. (a) Reconstructed lumen geometries of the LAD, LCX and RCA arteries. Red sections are reconstructed using a hybrid OCT/bi-plane angiography method to produce a lumen profile to a high accuracy. The blue sections represent the geometry reconstructed using bi-plane angiography. The adhered thrombus is defined by the green surface, extracted from OCT data. (b) Haemodynamic metrics extracted from CFD simulations. Time-Averaged Wall Shear Stress (TAWSS), Oscillatory Shear Index (OSI), Relative Residence Time (RRT) and Time-Averaged Wall Shear Stress Gradient (TAWSSG). Both ‘rest’ and ‘exercise’ flow rate conditions were simulated. The thrombus is the opaque portion of the metrics, whilst the remainder of the lumen is semi-transparent. Flow is from top to bottom for all images. Minimum and maximum values for the legends are the lower and upper quartiles of the respective metrics averaged across the rest and exercise cases separately, as shown in Table S4 & Table S5. RRT ranges are normalised in respect to the averaged median RRT at the ‘non-diseased’ location, with the median values being 1.11 and 0.43 for rest and exercise respectively. Ensight 10.2.3, was used to post-process and visualise the results.

| **Rest** | TAWSS (Pa) | | | TAWSSG (Pa/mm) | | WSS_Max_ (Pa) | |
| --- | --- | --- | --- | --- | --- | --- | --- |
|  | Non-diseased | Thrombus | Thrombus/  Non-diseased | Non-diseased | Thrombus | Non-diseased | Thrombus |
| Case 1 | 0.75 | 4.79 | 2.68 | 0.28 | 3.99 | 0.59 | 3.79 |
| Case 2 | 2.41 | 11.90 | 2.30 | 1.58 | 22.00 | 1.57 | 8.10 |
| Case 3 | 1.03 | 4.64 | 2.18 | 0.32 | 7.62 | 0.64 | 3.16 |
| Case 4 | 2.10 | 9.25 | 2.14 | 0.99 | 3.95 | 1.38 | 6.33 |
| Case 5 | 0.46 | 1.82 | 1.98 | 0.22 | 0.71 | 0.38 | 1.44 |
| Case 6 | 1.10 | 2.31 | 1.07 | 0.41 | 2.09 | 0.70 | 3.09 |
| Case 7 | 0.26 | 7.66 | 4.86 | 0.08 | 10.90 | 0.22 | 5.92 |
| Case 8 | 1.47 | 28.60 | 4.28 | 0.55 | 39.20 | 0.91 | 39.29 |
| Case 9 | 2.59 | 10.70 | 2.05 | 0.96 | 18.40 | 1.64 | 7.40 |
| Case 10 | 1.01 | 23.00 | 4.51 | 0.38 | 58.20 | 0.58 | 15.83 |
| Case 11 | 0.62 | 2.14 | 1.80 | 0.10 | 1.03 | 0.50 | 1.70 |
| Case 12 | 2.28 | 7.29 | 1.68 | 0.65 | 8.90 | 1.50 | 4.96 |
| Case 13 | 4.16 | 9.68 | 1.22 | 2.33 | 38.40 | 2.73 | 6.81 |
| Case 14 | 0.48 | 1.08 | 1.18 | 0.06 | 0.87 | 0.32 | 0.69 |
| Case 15 | 4.58 | 9.51 | 1.05 | 1.04 | 8.20 | 3.05 | 6.50 |
| Case 16 | 1.34 | 2.35 | 0.81 | 0.32 | 2.88 | 0.90 | 1.58 |
| Case 17 | 0.48 | 0.46 | -0.04 | 0.11 | 0.27 | 0.39 | 0.38 |
| Minimum | 0.26 | 0.46 | -0.04 | 0.06 | 0.27 | 0.22 | 0.38 |
| Quartile 1 | 0.62 | 2.31 | 1.18 | 0.22 | 2.09 | 0.49 | 1.70 |
| Median | 1.10 | 7.29 | 1.98 | 0.38 | 7.62 | 0.70 | 4.95 |
| Quartile 3 | 2.28 | 9.68 | 2.15 | 0.96 | 18.36 | 1.50 | 6.81 |
| Maximum | 4.58 | 28.57 | 4.86 | 2.33 | 58.22 | 3.05 | 39.29 |

| **Exercise** | TAWSS (Pa) | | | TAWSSG (Pa/mm) | | WSS_Max_ (Pa) | |
| --- | --- | --- | --- | --- | --- | --- | --- |
|  | Non-diseased | Thrombus | Thrombus/  Non-diseased | Non-diseased | Thrombus | Non-diseased | Thrombus |
| Case 1 | 1.93 | 12.00 | 2.65 | 0.87 | 10.40 | 5.27 | 9.98 |
| Case 2 | 7.89 | 33.40 | 2.08 | 5.93 | 69.40 | 1.40 | 1.59 |
| Case 3 | 3.11 | 12.30 | 1.99 | 1.16 | 21.90 | 13.40 | 54.50 |
| Case 4 | 6.74 | 25.80 | 1.94 | 3.77 | 12.70 | 9.92 | 32.54 |
| Case 5 | 1.08 | 4.59 | 2.10 | 0.57 | 1.93 | 11.44 | 41.51 |
| Case 6 | 2.93 | 5.63 | 0.94 | 1.24 | 6.62 | 0.86 | 30.72 |
| Case 7 | 0.59 | 20.40 | 5.11 | 0.21 | 32.30 | 19.00 | 43.90 |
| Case 8 | 3.85 | 76.50 | 4.31 | 1.65 | 118.00 | 4.73 | 8.61 |
| Case 9 | 8.35 | 27.30 | 1.71 | 4.13 | 55.20 | 2.25 | 7.94 |
| Case 10 | 2.34 | 62.60 | 4.74 | 1.03 | 171.00 | 5.25 | 20.30 |
| Case 11 | 1.51 | 5.33 | 1.82 | 0.29 | 2.96 | 0.59 | 3.79 |
| Case 12 | 6.16 | 20.00 | 1.70 | 1.92 | 28.90 | 15.18 | 44.03 |
| Case 13 | 11.20 | 28.10 | 1.33 | 7.20 | 119.00 | 1.57 | 6.85 |
| Case 14 | 1.03 | 2.57 | 1.31 | 0.20 | 2.34 | 3.63 | 104.42 |
| Case 15 | 11.80 | 26.20 | 1.15 | 3.06 | 26.00 | 1.58 | 4.04 |
| Case 16 | 3.33 | 6.14 | 0.88 | 0.96 | 9.02 | 6.25 | 125.07 |
| Case 17 | 1.00 | 1.07 | 0.11 | 0.28 | 0.71 | 18.27 | 48.17 |
| Minimum | 0.59 | 1.07 | 0.11 | 0.20 | 0.71 | 0.59 | 1.59 |
| Quartile 1 | 1.51 | 5.63 | 1.31 | 0.57 | 6.62 | 1.58 | 7.94 |
| Median | 3.11 | 19.96 | 1.82 | 1.16 | 21.86 | 5.25 | 30.72 |
| Quartile 3 | 6.74 | 27.29 | 2.02 | 3.06 | 55.19 | 11.44 | 44.03 |
| Maximum | 11.81 | 76.51 | 5.11 | 7.20 | 170.93 | 19.00 | 125.07 |

Table S4 - Area-averaged values of TAWSS, TAWSSG and WSS_Max_ (maximum flow during the cardiac cycle) at the site defined as being normal flow (non-diseased) and at the adhered thrombus location. Thrombus/non-disease ratio displayed as log2-fold change

| **Rest** | OSI (-) | | | RRT | |
| --- | --- | --- | --- | --- | --- |
|  | Non-diseased | Thrombus | Thrombus/  Non-diseased | Non-diseased (1/Pa) | Thrombus (-) |
| Case 1 | 4.90E-04 | 7.76E-06 | -5.98 | 1.70E+00 | -8.74E-01 |
| Case 2 | 2.73E-02 | 1.09E-03 | -4.65 | 1.45E+00 | -8.71E-01 |
| Case 3 | 1.67E-02 | 1.15E-02 | -0.55 | 1.91E+00 | -7.02E-01 |
| Case 4 | 6.10E-03 | 6.40E-05 | -6.57 | 7.13E-01 | -8.31E-01 |
| Case 5 | 4.96E-05 | 3.84E-05 | -0.37 | 2.31E+00 | -6.99E-01 |
| Case 6 | 1.26E-03 | 1.59E-04 | -2.98 | 1.11E+00 | -5.35E-01 |
| Case 7 | 4.02E-04 | 1.29E-03 | 1.69 | 4.06E+00 | -8.10E-01 |
| Case 8 | 5.89E-04 | 2.29E-04 | -1.36 | 7.55E-01 | -9.26E-01 |
| Case 9 | 1.17E-03 | 1.69E-03 | 0.53 | 4.75E-01 | -6.42E-01 |
| Case 10 | 2.07E-04 | 1.03E-02 | 5.64 | 1.06E+00 | -8.82E-01 |
| Case 11 | 3.58E-05 | 1.64E-04 | 2.20 | 1.64E+00 | -6.45E-01 |
| Case 12 | 5.97E-05 | 1.22E-03 | 4.35 | 4.54E-01 | -5.46E-01 |
| Case 13 | 1.32E-04 | 9.79E-03 | 6.21 | 2.66E-01 | 7.28E-01 |
| Case 14 | 1.51E-04 | 1.71E-02 | 6.83 | 2.18E+00 | 3.66E-01 |
| Case 15 | 1.20E-05 | 1.32E-02 | 10.10 | 2.35E-01 | 6.25E-01 |
| Case 16 | 7.56E-04 | 3.07E-02 | 5.34 | 8.26E-01 | 1.58E+00 |
| Case 17 | 1.64E-05 | 6.24E-05 | 1.92 | 2.21E+00 | 9.14E-02 |
| Minimum | 0.00E+00 | 0.00E+00 | -6.57 | 2.40E-01 | -9.30E-01 |
| Quartile 1 | 1.00E-04 | 2.00E-04 | -1.36 | 7.10E-01 | -8.30E-01 |
| Median | 4.00E-04 | 1.20E-03 | 1.69 | 1.11E+00 | -6.50E-01 |
| Quartile 3 | 1.20E-03 | 1.03E-02 | 5.42 | 1.91E+00 | 9.00E-02 |
| Maximum | 2.73E-02 | 3.07E-02 | 10.10 | 4.06E+00 | 1.58E+00 |

| **Exercise** | OSI (-) | | | RRT | |
| --- | --- | --- | --- | --- | --- |
|  | Non-diseased | Thrombus | Thrombus/  Non-diseased | Non-diseased (1/Pa) | Thrombus (-) |
| Case 1 | 7.84E-04 | 1.21E-05 | -6.02 | 7.57E-01 | -8.87E-01 |
| Case 2 | 1.10E-02 | 2.13E-03 | -2.37 | 3.81E-01 | -7.85E-01 |
| Case 3 | 8.18E-03 | 1.13E-02 | 0.47 | 5.56E-01 | -5.45E-01 |
| Case 4 | 4.71E-03 | 4.67E-05 | -6.66 | 2.62E-01 | -8.30E-01 |
| Case 5 | 1.41E-04 | 6.38E-05 | -1.15 | 1.03E+00 | -7.02E-01 |
| Case 6 | 9.21E-04 | 4.95E-04 | -0.90 | 4.26E-01 | -4.42E-01 |
| Case 7 | 1.38E-03 | 5.64E-04 | -1.29 | 1.95E+00 | -8.55E-01 |
| Case 8 | 2.84E-04 | 7.89E-03 | 4.79 | 3.07E-01 | -8.70E-01 |
| Case 9 | 3.55E-03 | 5.13E-03 | 0.53 | 1.66E-01 | -4.59E-01 |
| Case 10 | 2.28E-04 | 1.09E-02 | 5.57 | 4.82E-01 | -9.15E-01 |
| Case 11 | 4.15E-05 | 6.36E-04 | 3.94 | 6.73E-01 | -6.07E-01 |
| Case 12 | 2.97E-05 | 3.07E-03 | 6.69 | 1.68E-01 | -4.01E-01 |
| Case 13 | 8.23E-05 | 7.98E-03 | 6.60 | 1.03E-01 | 5.49E-01 |
| Case 14 | 1.28E-02 | 2.85E-02 | 1.16 | 1.33E+00 | 2.33E-01 |
| Case 15 | 8.35E-06 | 9.53E-03 | 10.16 | 9.59E-02 | 1.40E+00 |
| Case 16 | 4.82E-03 | 1.36E-02 | 1.49 | 3.78E-01 | 1.52E+00 |
| Case 17 | 4.46E-05 | 1.26E-04 | 1.50 | 1.11E+00 | -2.09E-02 |
| Minimum | 0.00E+00 | 0.00E+00 | -6.66 | 1.00E-01 | -9.10E-01 |
| Quartile 1 | 1.00E-04 | 5.00E-04 | -1.15 | 2.60E-01 | -8.30E-01 |
| Median | 8.00E-04 | 3.10E-03 | 1.16 | 4.30E-01 | -5.40E-01 |
| Quartile 3 | 4.70E-03 | 9.50E-03 | 4.99 | 7.60E-01 | -2.00E-02 |
| Maximum | 1.28E-02 | 2.85E-02 | 10.16 | 1.95E+00 | 1.52E+00 |

Table S5 - Area-averaged values of OSI and RRT at the site defined as being normal flow (non-diseased) and at the adhered thrombus location. Thrombus/non-disease ratio displayed as log2-fold change
